# Delving Deep into Multiscale Pedestrian Detection via Single Scale Feature Maps

**DOI:** 10.3390/s18041063

**Published:** 2018-04-02

**Authors:** Xinchuan Fu, Rui Yu, Weinan Zhang, Jie Wu, Shihai Shao

**Affiliations:** 1 National Key Laboratory of Science and Technology on Communications, University of Electronic Science and Technology of China, Chengdu 611731, China; ssh@uestc.edu.cn; 2 Department of Computer Science, University College London, London WC1E 6BT, UK; r.yu@cs.ucl.ac.uk; 3 Department of Computer Science & Engineering, Shanghai Jiao Tong University, Shanghai 200240, China; wnzhang@sjtu.edu.cn; 4 Department of MOE Research Center for Software/Hardware Co-Design Engineering and Application, East China Normal University, Shanghai 200062, China; 52151500020@stu.ecnu.edu.cn

**Keywords:** pedestrian detection, boosted decision tree, scale invariance, receptive field correspondence, soft decision tree

## Abstract

The standard pipeline in pedestrian detection is sliding a pedestrian model on an image feature pyramid to detect pedestrians of different scales. In this pipeline, feature pyramid construction is time consuming and becomes the bottleneck for fast detection. Recently, a method called multiresolution filtered channels (MRFC) was proposed which only used single scale feature maps to achieve fast detection. However, there are two shortcomings in MRFC which limit its accuracy. One is that the receptive field correspondence in different scales is weak. Another is that the features used are not scale invariance. In this paper, two solutions are proposed to tackle with the two shortcomings respectively. Specifically, scale-aware pooling is proposed to make a better receptive field correspondence, and soft decision tree is proposed to relive scale variance problem. When coupled with efficient sliding window classification strategy, our detector achieves fast detecting speed at the same time with state-of-the-art accuracy.

## 1. Introduction

Pedestrian detection aims to locate all the pedestrians in an image. It has many real world applications, such as driving assistance and video surveillance. It also serves as a playground for many image processing and machine learning algorithms. There have been well established benchmark datasets [1,2,3,4] and a variety of methods have published to address this problem [3,5,6,7,8,9,10]. In many real applications, detection speed is often as important as accuracy, like in Advanced Driver Assistance Systems (ADAS) [11]. Although recently deep learning methods have achieved the state-of-the-art accuracy in pedestrian detection, the detection speed is often low even with high-end GPU [12,13]. On the other hand, boosted decision tree (BDT) methods remain highly competitive in this area for its efficacy with (light-weight) CPU implementation [14,15,16]. In this paper, we focus on the BDT methods for pedestrian detection.

Pedestrians in images may exhibit a large range of scale, which constitutes a significant mode of intra-class variability. For example, the heights of the pedestrian samples in the Caltech pedestrian dataset [1] range from 7 to 476 pixels. How to detect pedestrians of different scales becomes a key problem in pedestrian detection.

It is obvious that pedestrians of different scales have different representations in the original image—at least they have different numbers of pixels. A feature will be appropriate for multiscale detection if it is invariant across different scales. Thus for a long time, the community of detection and recognition strives for building the so-called scale-invariant representations. Unfortunately, many features are not scale invariant, including Histograms of Oriented Gradients (HOG) [3] which are widely used in pedestrian detection. This means a feature extracted in a large scale pedestrian is different from a corresponding feature extracted in a small scale pedestrian. Therefore, to achieve scale-invariance, the standard pipeline in pedestrian detection is to construct an image pyramid, compute feature maps at each layer of the pyramid, and finally perform sliding window detection with a trained pedestrian model. As the pedestrian scale exhibits a large scope, the pyramid need to contain many layers and the construction of feature pyramid takes a lot of time, which becomes the bottleneck for fast pedestrian detection.

Could we avoid feature pyramid construction and detecting multiscale pedestrians only using single scale feature maps while still achieve high accuracy? In this paper, we will delve deep into this problem. In fact, some pioneer works [14,17] have shown promising results in this direction. Our work is based on MRFC [14] which is the most recent work on this topic. We analyze some weaknesses of their work and show how to make improvements to it. The main contributions of our paper are as follows:

1. To achieve better receptive field correspondence, we propose to use scale-aware pooling instead of gridwise sampling. Based on it, we use efficient difference integral channels (DICs) to enrich our features.
2. To relieve the scale variance problem, we propose to use a soft decision tree to build a weak classifier in the BDT cascade. Two branches of the soft decision tree specialize in large or small scale instances respectively.
3. Experiments show that when coupled with efficient sliding window classification strategy, our method achieves state of the art accuracy with fast detection speed.

A preliminary version of this work appeared in [18]. The main extension in this paper is as follows. Firstly, we add the content about soft decision tree (Section 4) and sparse grid detection (Section 7). Secondly, motion channels are added to our detector in this paper. Thirdly, more experiment results are given. For example, all the experiments on KITTI dataset [2], the tables listing the performance on different setups of our method and plots which show the performance of our detector under conditions of small scale, atypical aspect ratio and partial occlusion are newly added. Fourthly, on Caltech dataset [1], we achieve a noticeable accuracy improvement compared to that in [18] (average miss rate from 15.89% to 12.96%).

The rest of this paper is organized as follows. We first give a review of the related work of our paper in Section 2. Then based on the shortcomings of MRFC, we describe how to make a better receptive field correspondence using scale-aware pooling in Section 3, how to relive the scale variance problem using soft decision tree in Section 4. To accelerate the speed in the sliding window classification stage, we propose two strategies in Section 5. Experiment results are given in Section 6. Finally, we conclude in Section 7.

## 2. Related Work

Features play a key role in pedestrian detection [19]. The first popular features for pedestrian detection are HOG features. Many pedestrian and general object detectors adopt these features [20,21,22,23]. Based on HOG features, Dollár et al. [24] proposed to use LUV color channels, gradient magnitude channel and 6 orientation channels as feature maps. Since then, these 10 feature maps have become popular and a lot of recent papers are based on these feature maps [5,6,14,25,26,27]. For its effectiveness and efficiency, many deep learning methods also adopt them to perform region proposal [28,29,30].

**Figure 1:**
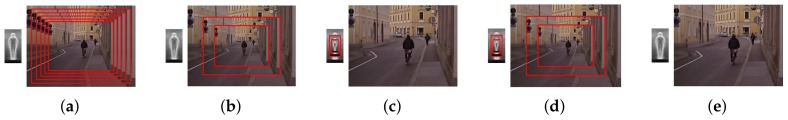
Different multiscale detection strategies. (**a**) Dense image pyramid and single classifier; (**b**) Sparse image pyramid and single classifier; (**c**) Single image scale and sparse classifier pyramid; (**d**) Sparse image pyramid and sparse classifier pyramid; (**e**) Single image scale and single classifier.

Multiscale detection methods usually aim to find a scale-invariant representation. If the features used are scale-invariant, the detection can be performed using single scale feature maps and multiscale objects are detected by resizing the model [31]. Unfortunately, not all features used in pedestrian detection are scale-invariant. As to the 10 channels we described above, the LUV color channels are scale invariant, but the gradient magnitude channel and the 6 orientation channels are not scale invariant. In such cases, the standard treatment is to use dense image pyramid as shown in Figure 1a, which results in high computational cost. Some papers are published to accelerate this process. FPDW [32] computed a sparse feature pyramid and approximated intermediate feature scales using the exponential scaling law (Figure 1b). In practice, this strategy usually leads to noticeable accuracy degradation. Instead of using feature pyramid, VeryFast [33] trained a sparse classifier pyramid according to different pedestrian scales and approximated intermediate classifier scales using the exponential scaling law (Figure 1c). This strategy actually transforms testing time to training time. FastCF [16] used both feature pyramid and classifier pyramid (Figure 1d).

Recently, another point of view [14,17] was proposed which ignored the requirement for scale invariance. The authors of these papers argued that though there are many sources of intra-class variance for pedestrian detection, like illumination, orientation and occlusion, et al., which lead to significant different representation for the pedestrian class, a BDT is able to handle this intra-class variance and provide a good result. They thought that the feature variance caused by different scales is just another source of intra-class variance and will hopefully be handled well by the BDT. The precondition is that the number of weak classifiers and the training data is large enough. Based on this idea, they just used a single scale feature maps and trained one pedestrian model (Figure 1e). At testing time, they scaned these feature maps with resized pedestrian models.

We adopt this point of view, and our work makes some improvements in comparison with previous works. WordChannels [17] uses 192 feature maps, for each one of which an integral map is computed, which results in a high computational cost. The authors used GPU to implement their algorithm. MRFC [14] is a more recent work from the same authors which achieves fast detection based on CPU. It computed 210 feature maps efficiently without integral map computing. The problem of this method is the features’ receptive field correspondence (which will be described in detail in Section 3) between different scales is weak, which limits the accuracy of the method. To make a more accurate receptive field correspondence, we borrow the idea from spatial pyramid pooling (SPP) [34], also known as spatial pyramid matching (SPM) [35]. It partitions the feature map into a predefined number of divisions and performs pooling in each division. Thus feature maps of various sizes are converted into a fixed length vector. This technique is very useful in convolutional neural network (CNN)-based detectors as the fully connected layer needs to be fed in a vector with fixed length. It has become a key component in fast-RCNN [36] and faster-RCNN [37] detectors. While the CNN use max-pooling, we use average-pooling as it can be computed efficiently via integral maps [31].

Both [14,17] ignored the requirement for scale invariance. This is another problem of their strategy. Though their strategy will work, the scale variance problem will undoubtedly degrade performance. In this paper, we relieve the scale variance problem using a divide and conquer strategy for different scales. Some work explicitly models the differences of different scales. Rajaram et al. [38] trained different models for different scales and at test time the detection results were combined. Yan et al. [39] extended the idea to the popular Deformable Part Models (DPM) detector [22], which applied two resolution-aware transformations $P_{H}$ and $P_{L}$ for high and low resolution samples respectively. Park et al. [40] shared the low resolution model for all samples, and for large-scale samples the high resolution model was added. The idea of [28] is close to ours, which uses two built-in subnetworks to detect pedestrians from different scales. While their model is used for CNN, we adapt this idea to BDT by using soft decision tree.

## 3. Scale-Aware Pooling

Our work is inspired by MRFC. To our knowledge, it is the first CPU-based solution which only uses a single scale feature maps and a single pedestrian model. In this section, we first give a simple description of the basic idea of MRFC, then explain its weakness in receptive field correspondence and show how to improve it by scale-aware pooling.

**Figure 2:**
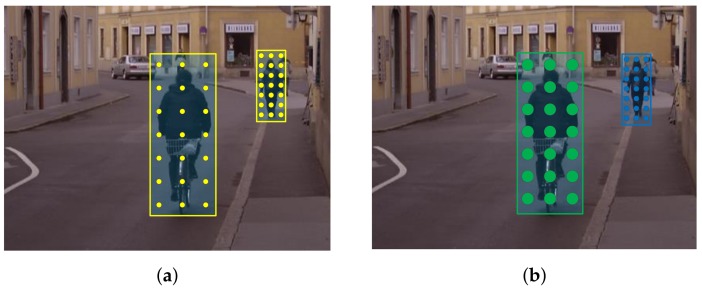
Illustration of the problem of receptive field correspondence in MRFC method. (**a**) The yellow circles represent the receptive fields of a feature. Note for pedestrians of different scales, the area of the circle do not change, which is unreasonable; (**b**) The ideal circumstance is the receptive field of a feature resizes according to the scale of the pedestrian. Note the areas are different for circles of different colors.

The base feature maps used in MRFC are the 10 LUV+HOG channels we describe in Section 2. After the 10 channels are computed for the original image, a 3×3 box filter is applied sequentially to the original 10 channels 6 times, obtaining 70 channels. This process can also be taken as applying 6 convolution kernels with different standard deviation σ to the original 10 channels. Then two edge filters (vertical and horizontal) are applied to each of these channels, yielding 210 channels in total, which are the so called multiresolution filtered channels. To detect pedestrians at multiple scales, pedestrian models of different scales are slid on the computed channels. The classification features are extracted by sampling from the channels in a gridwise manner and the space between grids are adapted to the window size, as illustrated in Figure 2a. In this way, the same number of features is obtained for pedestrians of different sizes. At training time, as opposed to resizing the pedestrian to a fixed size like traditional methods, the features are extracted from the original pedestrian size in the image. At testing time, there is no need to compute an image pyramid for multiscale detection. The 210 channels could be computed very efficiently. Moreover, after the multiresolution filtered channels are computed, the feature accessing only needs a single pixel indexing like ACF detector [5]. Thus the speed of the detector is very fast.

However, there are some shortcomings of the original implementation of the MRFC method. One of them is about the receptive field correspondence. As we stated above, there are totally 7 different receptive field sizes in these channels. The problem is that these 7 sizes are fixed. As shown in Figure 2a, in MRFC’s implementation, the receptive field of a feature does not change with the size of pedestrians. Thus a feature’s receptive field in a small pedestrian does not correspond to that in a large pedestrian. In this circumstances a feature corresponding to the nose for a large pedestrian may correspond to the whole face for a small pedestrian, which is unreasonable. The ideal circumstance is that the receptive field of a feature resizes along with the scale of the pedestrian, as shown in Figure 2b.

**Figure 3:**
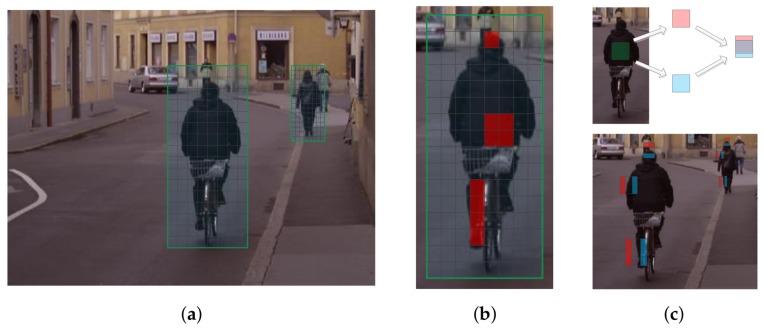
Illustration of the feature extraction process of our method. (**a**) Feature maps of different scales are divided into the same number of cells whose size vary with the pedestrian size; (**b**) Features are extracted by average pooling in different regions which are composed of one or more cells; (**c**) Top: Pooling in feature gradient maps is equivalent to computing difference of two shifted pooling regions, which has similar effect with Non-Neighboring Features (NNF). Down: Some discriminative features in DICs.

Now we show how to overcome this problem. Similar to SPP, we partition the detection window to m×n cells, and a feature is computed by average pooling in one or more cells, as illustrated in Figure 3b. In this way, the area of a cell is resized according to the size of the detection window which leads to a better correspondence of features’ receptive field. In our work, we use 23×11 cells and the pooling region is constrained to be not larger than a 4×2 cell, which yields 1806 features for each feature map. In MRFC, 7 receptive field sizes are formed by sequentially convolution. In our method, 8 different types of receptive field are formed by combining the basic cells with their areas varying according to the window size.

Our baseline implementation also uses the 10 channels and the 20 gradient channels, resulting in 30 feature maps in total. Note unlike other types of convolutional feature maps, the feature gradient maps can be computed very efficiently by using SSE (Streaming SIMD Extensions) instructions. In our experiment, we also test adding motion channels using the method described in [41], which result in another 3 channels (1 base channel and 2 gradient channels). Adding motion channels will increase accuracy, but will significantly slow down detection speed, as shown in Section 6.

**Figure 4:**
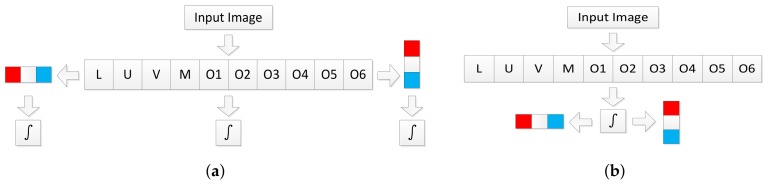
Acceleration strategy for computing integral maps. (**a**) Naive approach. Every feature maps need to be integrated; (**b**) Our method. Only the original 10 feature maps need to be integrated.

To quickly perform average pooling, integral maps [31] are pre-computed before detection, then the sum of feature values in a region will be computed in constant time irrelevant to the region size. The naive implementation computes an integral map for each of the 30 feature maps (10 base feature maps + 20 gradient feature maps), as shown in Figure 4a. However, consider the commutation law for convolution, we have

(1) $$\begin{array}{c} \Omega (x,y)*G(x,y)*u(x,y)=\Omega (x,y)*u(x,y)*G(x,y)\;, \end{array}$$

where Ω is the feature map, G(x,y) is the gradient filter and u(x,y) is the step function. Note that integration could be taken as convolution with the step function. We only need to compute the integral maps of the original 10 feature maps and then compute 20 gradient maps on the computed integral map, as shown in Figure 4b. We call these channels difference integral channels (DICs). In addition to gradient filters, this strategy could also be used for other linear filters. Features formed by nonlinear transformations, like word channels [17] or CNN based features (after ReLU layer), can not take such an advantage.

In fact, the features extracted in DICs (Figure 3c) resemble the Non-Neighboring Features (NNF) [27] which are demonstrated to be effective for pedestrian detection. NNF are differences of non-neighboring rectangular areas in the same horizontal. Our DICs are superior to NNF in the following aspects. First, NNF use a fixed model size, so an image pyramid is required, whereas we do not construct image pyramid, which saves a lot of computation. Second, accessing a NNF feature needs to compute average pooling for two regions, whereas in our method, we only need to perform average pooling for one region. Third, the NNF implementation only considers two regions in the same horizontal, whereas we also include the two regions in the same vertical which may be useful to represent some pedestrian structure like head, shoulder, and feet. Figure 3c shows some discriminative features in DICs.

By definition, an average pooling feature is computed by first sum all the feature values in the region, then divided by the region size. For efficiency, we do not perform the dividing operation at test time. Instead, we change the threshold of the decision stumps in each decision tree in advance. That is to say, though we only train one detector, we switch this detector to *n* detectors where *n* is the number of the template scales. These detectors have the same feature indexes but with different thresholds which are multiplied by their corresponding region size.

## 4. Soft Decision Tree

The authors of MRFC ignore the requirement for scale invariance and argue that the BDT will handle the intra-class variance caused by using features which are not scale invariant. The experiments in [14] show the feasibility of this idea. However, this strategy undoubtedly leads to a more diverse feature distribution and the decision surface between positive and negative samples become more complex. This leaves a more difficult classification task to the BDT cascade. Hence we believe if we could relive the scale variance problem to some extent, a better result is expected.

A naive solution is to using different models for each scale, which is adopted in [33]. This leads to a more training expense. Furthermore, there is a dilemma in how to use the training data. If we train a specific model using samples of its corresponding scale, we need to split the training data for each scale which leads to insufficient training data. On the other hand, if we train each model using all the samples by resizing all the samples to the corresponding scale, the blurring artifacts caused by the resizing operation [33] leads to unreliable training data.

Here we propose to use a soft decision tree [42] to build each weak classifier in the BDT, where the two branches of the tree specialize in large and small scale pedestrians respectively. In this solution all the samples are involved in training in a single BDT cascade.

**Figure 5:**
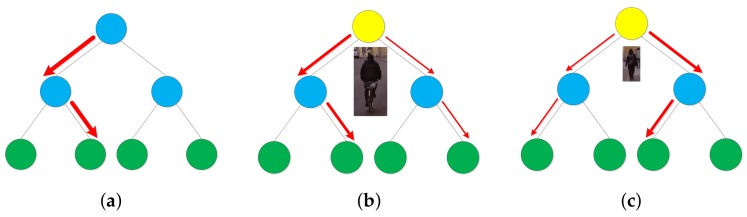
Comparison between hard decision trees and soft decision trees. The blue, yellow and green nodes denote hard decision node, soft decision node and leaf nodes respectively. The red arrows denote the flow of sample weights. (**a**) The hard decision tree is composed of hard decision nodes and leaf nodes. For a given sample, the hard decision node direct all its weight to one of its children; (**b**) The root node of the soft decision tree is a soft decision node which directs the sample weight to both its children according to the sample size. Given a large sample, the soft decision node directs more weight to its left branch. Note the arrow of the left branch is thicker than the arrow of the right branch; (**c**) Another example of the soft decision tree with a small sample. The soft decision node directs more weight to its right branch.

While a hard decision node used in hard decision trees deterministically directs a sample *x* to one of its children, a soft decision node directs a sample *x* to both its left and right branches with probabilities P(L|x) and P(R|x) and the output of the soft decision node is computed by weighted sum of the output of its two branches, as shown in Figure 5. The soft decision tree in [42] use soft decision node for all the non-leaf nodes of the tree. In testing time, every instance must pass through all the intermediate nodes until it reaches the leaf nodes. This scheme is only suitable for a single decision tree. For BDT with thousands of trees, this scheme leads to high computation cost. Thus we simplify it by only using the soft decision node for the root node. As we are dealing with the multiscale detection problem, we make the left branch responsible for large scale pedestrians’ classification and the right branch responsible for small scale pedestrians’ classification. The following gate function is used to define the probabilities:

(2) $$\begin{array}{cc} P(L|x) & =\frac{1}{1+\exp[\frac{\bar{h}\;-\;h_{x}}{\beta }]} \end{array}$$

(3) $$\begin{array}{cc} P(R|x) & =1-P(L|x)\;, \end{array}$$

where $\bar{h}$ is the mean of pedestrian heights in the training set, $h_{x}$ is the height of the sample *x*, β is a hyper parameter which control the amount of smoothing for the gate function. As $h_{x}$ increases, P(L|x) gets larger and sample *x* direct more weight to the left branch. β reflects the degree of similarity of different scale pedestrians. When β is large, the function P(L|x) is gentle which means we think different scale instances are very similar. The extreme case is β→∞ which means we treat instances of all scales equally and the tree becomes an average ensemble. when β is small, the function P(L|x) is steep which means we think different scale instances have very different representation and need to be tackled differently. The extreme case is β→0 and the node becomes a hard decision node.

After the sample *x* is directed to both branches of the root node, it is evaluated by the two branches to get P(y=1|x,L) and P(y=1|x,R) where y∈{−1,+1} is the sample label. Then, the probability of *x* to be a positive sample given by the whole soft decision tree is

(4) $$\begin{array}{c} P(y=1|x)=P(L|x)P(y=1|x,L)+P(R|x)P(y=1|x,R)\;. \end{array}$$

Note that we use RealBoost [43] to train our BDT in which the leaf node do not outputs the probability, but the half log ratio

(5) $$\begin{array}{c} f\left(x\right)=\frac{1}{2}\log\frac{p(x)}{1-p(x)}\;, \end{array}$$

where p(x) is fraction of the positive sample weight in the leaf node. To get P(y=1|x,L) and P(y=1|x,R) in Equation (4), we need to switch the f(x) to its corresponding probability by the inverse function of Equation (5)

(6) $$\begin{array}{c} p\left(x\right)=\frac{e^{2f(x)}}{1+e^{2f(x)}}\;. \end{array}$$

Because in Realboost’s updating rule each weak classifier should output half log ratio, after we get P(y=1|x) using Equation (4), we need to switch it to its corresponding half log ratio using Equation (5). At training time, when the half log ratio is acquired for every sample, the sample weights are updated as the commonly used RealBoost.

At testing time, we need to compute Equation (6) two times for each soft decision tree, one for each branch. For efficiency, we switch the half log ratio to its corresponding probability using Equation (6) for all the tree nodes after training. By doing this, we avoid computing Equation (6) at testing time.

## 5. Accelerating Sliding Window Classifications

The advantage of using a single scale feature maps is that compared with traditional method which construct a dense pyramid, it greatly saves computation cost in the feature maps computation stage. However, apart from feature maps computation, there is another time consuming process in pedestrian detection: sliding windows classification. Because our method is based on regional average pooling, computation cost is higher at this stage compared to some other detectors like ACF [5] and LDCF [25]. For these detectors, after the feature pyramid is constructed, accessing a feature by a tree node only needs one pixel accessing, while for our method, it needs 4 pixel accessing and 3 plus/minus operation. Furthermore, the computation cost is doubled by our soft decision tree. Thus our single scale feature maps strategy must be followed by a efficient sliding window classification strategy, otherwise its advantage is limited because the later one will dominate the computation time. Luckily, based on the characteristics of pedestrian detection problem, there are various ways to accelerate this stage [14,44,45]. To demonstrate the advantage of using single scale feature maps, in this section we introduce two simple strategies to accelerate sliding window classification which will be used in our experiments.

### 5.1. Ground Plane Constraint

**Figure 6:**
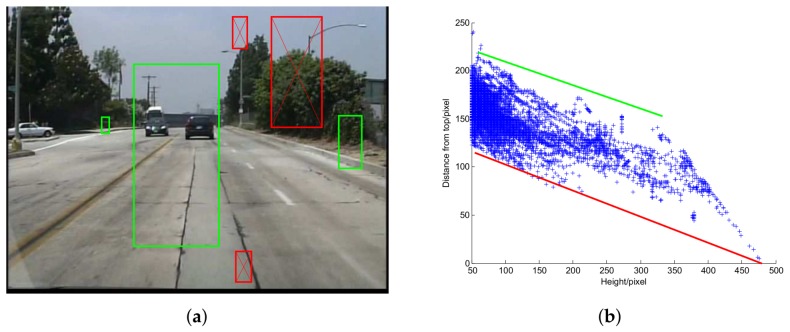
Illustration of GPC. (**a**) A pedestrian may be bounded by the green boxes, but may not be bounded by the red boxes; (**b**) (h,y)s of the pedestrian windows in the Caltech training set. They can be bounded by two straight lines.

For images captured by a fixed in-vehicle camera, pedestrians of a certain scale will not appear in some positions because of the ground plane constraint (GPC) [19], as shown in Figure 6a.

The key idea of GPC is that under some assumptions [40] which are valid for an in-vehicle camera, the projected height *h* and the vertical position *y* of a pedestrian exhibit a linear relationship. GPC has been widely used for pedestrian detection [14,15,23,40,46,47]. There are different ways to use GPC. Some of them adopt a post processing strategy using support vector machine (SVM) [15,40]. This type of methods aims to increase the detection accuracy, but lead to additional computation cost. Since our purpose is to accelerate sliding window classification, this type of methods is not suitable. Here we adopt the method proposed in our previous work [48] in which the possible position (h,y) of a pedestrian is bounded by two straight lines, as shown in Figure 6b. At detection stage, we only scan the possible pedestrian positions hence save computation cost. This method also has positive impact on accuracy for it gets rid of some false positives. Of course, there still exists some risk that in some special cases, some true positives in the testing set are not bounded by the two lines. In practice, we found the positive impact dominates.

### 5.2. Sparse Grid Detection

For a 480×640 image, there are more than 330,000 candidate windows (different scales and different positions) to be classified and most of them belong to background area. Note what we really want is the peak score windows which is the window with the local maximum score. Any windows with lower detection score in its neighbourhood will be suppressed by the peak score window after non-maximum suppression (NMS). To save computation cost, there is no need to evaluate all the candidate windows. We only need to make sure that all the peak score windows are evaluated.

Because the detector responses at nearby locations are correlated, the neighbouring positions of the peak score window usually also have positive responses. In other words, there exists a region of support (ROS) of the peak score window. For BDT cascade, the ROS size decreases with the number of the weak classifiers [44].

**Figure 7:**
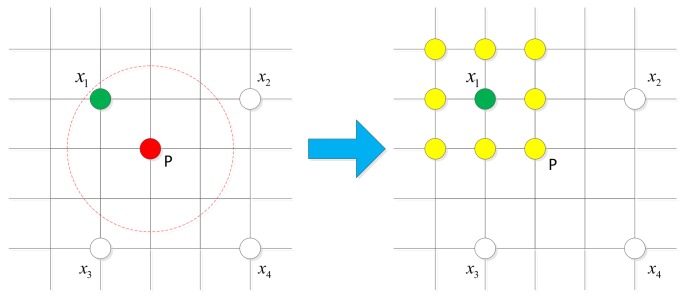
The sparse grid detection strategy. We begin by evaluate only a sparse grid ($x_{1},x_{2},x_{3},x_{4}$). Suppose *P* is a peak score window and its ROS is represented by the red dash line circle. Window $x_{1}$ is in the ROS, thus it will passes *k* stages of the BDT cascade and every window in its 3×3 neighbourhood is triggered (yellow circles).

Based on this, we begin by evaluate only a sparse grid $G_{3}$ with a step size of 3. If a window in $G_{3}$ passes *k* stages of the cascade, every window in its 3×3 neighbourhood is triggered to be evaluated. The reason behind this strategy is illustrated in Figure 7. Suppose the window *P* is a peak score window but not belongs to $G_{3}$, there will be a window $x_{1}\in G_{3}$ in its 3×3 neighbourhood. Window $x_{1}$ tends to have a positive score because of ROS and *P* will be triggered by $x_{1}$. Because $G_{3}$ only account for about 1/9 of all the sliding windows and the number of triggered windows tends to be very small, computation cost is greatly reduced.

When the *k* becomes larger, the ROS becomes smaller, the triggered windows become less and the detection speed becomes higher. However, this will increase the probability of missing peak score windows and degrade accuracy. In our experiment, we set k=20 which achieves a good tradeoff between speed and accuracy.

## 6. Experiments

In this section, we evaluate our proposed method on two standard pedestrian detection datasets: KITTI and Caltech. They are currently the most popular and widely used ones in the literature.

A detected bounding box ($bb_{d}$) is taken as a true positive if the Intersection-over-Union (IoU) with a groundtruth bounding box ($bb_{g}$) is greater than a threshold. The IoU is defined as

(7) $$\begin{array}{c} \mathrm{IoU}\left(bb_{d},bb_{g}\right)=\frac{bb_{d}⋂bb_{g}}{bb_{d}⋃bb_{g}}\;, \end{array}$$

and for both these two benchmarks, the IoU threshold is set to 0.5. Results on Caltech are compared using miss rate vs. False-Positive-Per-Image (FPPI) curves, which is the well-recognized evaluation metric for pedestrian detection [49]. Methods are ranked by log average miss rate (MR) which is computed by averaging miss rate at 9 FPPI points that are evenly spaced in the log-space ranging from $10^{-2}$ to $10^{0}$. Results on KITTI are compared using precision-recall curves, and methods are ranked by the average precision (AP) at 11 evenly spaced recall points ranging from 0 to 1.

### 6.1. Experiments on KITTI Dataset

The KITTI object detection benchmark has 7481 training and 7518 test images. It contains three object classes for evaluation: Car, Pedestrian, and Cyclist. Here we only choose pedestrian class for evaluation. KITTI differentiates the difficulty in identifying pedestrians to three levels: easy, moderate and hard, corresponding to different height, occlusion and truncation. Methods are ranked based on the moderate difficult level (the minimum height of bounding box is 25 pixels, the maximum occlusion level is “partly occluded” and the maximum truncation is 0.30).

In order to tune parameter and analysis the impact of different components of our algorithm, we split the training set into training and validation sets. As in [38], we ensure the images of the training and validation sets come from different video sequences and the number of images and pedestrians are comparable. As a result, the training set has 3740 images with 1792 pedestrians and the validation set has 3741 images with 1791 pedestrians. According to the evaluation metric, we need to detect pedestrians taller than 25 pixels. The smallest model size is set as 32×16 (including some background area) and we use 10 scales per octave. The sliding window stride is set to 1/16 of the window height/width in the vertical/horizontal direction. The final classifier is built via three rounds of hard negative mining (starting from a forest with 32 trees, and then 256, 1024, 4096 trees). Realboost [43] are used to train our model and the weak classifiers are level-4 decision trees. In the last round, we switch our RealBoost algorithm to the shrinkage version as is used in [29,50]. The shrinkage parameter is set to 0.5.

As stated in Section 4, the parameter β control the amount of smoothing of the gate function. We first test the effect of different β values. The result is listed in Table 1. From this table we see β=50 performs best, hence in the following experiment we set β=50.

Next we evaluate the impact of the five different components of our algorithm on accuracy and speed. The speed is tested on a single core of Intel i7 6700K CPU (4 GHz) and is measured as frames per second (FPS). To simplify notation, we use the following abbreviation: scale-aware pooling (SAP), soft decision tree (SDT), ground plane constraint (GPC) and sparse grid detection (SGD). Table 2 shows the impact of different combinations of the five components. We divide the five components into two categories. One category includes SAP, SDT and motion features, which are used for improving accuracy. Another category includes GPC and SGD, which are used for accelerating detection. Any AP/FPS value in the table corresponds to a detector combining some components for accuracy and some components for speed. For example, the AP/FPS value in the last row and the third column (70.53/1.65) denotes the performance of the detector combining SAP, SGD, motion features and GPC. Note SAP serves as our baseline detector, thus all the combinations in the table include SAP.

From this table, we could easily see the impact of different components by comparing the accuracy and speed between rows or columns. For example, By comparing the second and the third rows, we see that using soft decision tree definitely improve accuracy, but decrease speed. This is because for a hard decision tree, a sample is only directed to one branch of the root node, while for a soft decision tree, a sample is directed to both branches of the root node, hence the computation cost is doubled. By comparing the third and the forth rows, we see that adding motion features has the same effect: improving accuracy and decreasing speed. By comparing the second and the third columns, we see that though we use GPC to accelerate detection, it also improves accuracy for it gets rid of some false positives. By comparing the third and the forth columns, we see that using sparse grid evaluation significantly accelerates detection and slightly decreases accuracy. The fast version of our algorithm (67.29%/6.85FPS) is combining SAP, GPC and SGD, while combining SAP, SDT, motion features and GPC achieves the highest AP (70.53%/1.65FPS).

**Figure 8:**
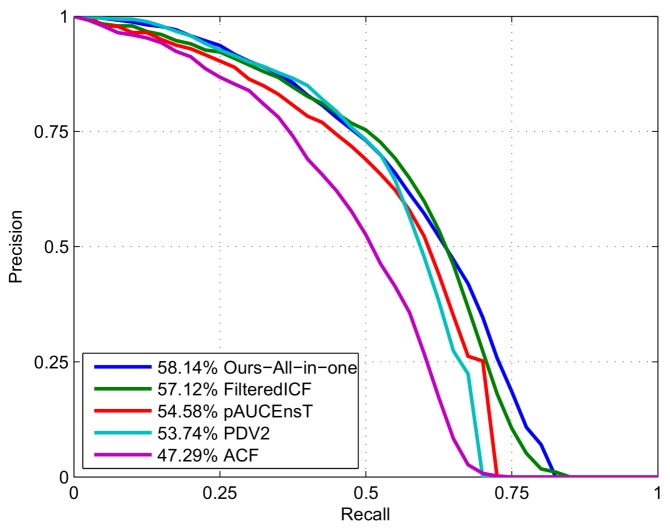
Comparison with non deep learning methods on the KITTI dataset. Our method does not achieve the highest precision for the whole recall range, but based on AP, our method outperforms the other methods.

To test our method on the test set, we train another model using the whole training set and all the components of our algorithm. Because of more training data, level-5 decision tree is used and the resultant detector is a little slower (3.37 fps) than the validation version. We use this model to detect pedestrians in the test images. Because the annotations of the test set are not public, the detection result is submitted to the KITTI evaluation server to get the evaluation result. We compare our method with some state-of-the-art non deep learning methods (which are listed on the KITTI benchmark website http://www.cvlibs.net/datasets/kitti/), as shown in Figure 8. In the precision-recall plot, for each recall point, the precision is the higher the better. From the figure, we see that our method does not achieve the highest precision for the whole recall range. As we stated at the beginning of this section, the KITTI benchmark use AP to rank different methods. The AP values are given at the figure legend. According to the AP values, our method outperforms all the other methods.

### 6.2. Experiments on Caltech Dataset

Caltech pedestrian datasets is currently the largest and the most widely used pedestrian detection dataset. It is more suitable for our method, because the amount of training data is much larger than that of KITTI dataset. It enables a comparison among more than 60 state-of-the-art approaches published during recent years. It consists of 250,000 labeled 640×480 frames (in 137 approximately minute long segments) which are divided into 11 sessions. The first 6 sessions are used for training and the last 5 sessions are used for testing. The standard evaluation is performed on every 30th frame of the test set, which yields 4024 images in all. Unless otherwise noted, the results are evaluated using the reasonable difficulty which means the pedestrian is at least 50 pixels in height with a visibility of at least 65%.

Following [25], our training images are collected by sampling one image out of every 4 consecutive frames. Because the evaluation metric only needs to detect pedestrians taller than 50 pixels, the smallest model size is set as 64×32. For efficiency, we downsample the channel by 2×, thus the feature map size of the smallest model is 32×16. Since there is more training data, we use twice the weak classifier number in each bootstrapping round (that is, starting from a model with 64 trees, and then 512, 2048, 8196 trees).

We compare our method with four representative methods (which are listed on the Caltech benchmark website http://www.vision.caltech.edu/Image_Datasets/CaltechPedestrians/): WordChannels [17], MRFC+Semantic [14], LDCF++ [15] and SDS-RCNN [13]. SDS-RCNN and LDCF++ are currently the best deep learning and non-deep learning methods reported on Caltech benchmark respectively. WordChannels and MRFC+Semantic are another two methods which also based on single scale feature maps like our method. MRFC+Semantic is the MRFC detector enhanced by semantic segmentation channels which need extra data set to train. The original MRFC achieves a MR of 19.09% with a speed of 20FPS. MRFC+Semantic achieves higher accuracy (16.84%) but slow down detection speed (8FPS).

**Figure 9:**
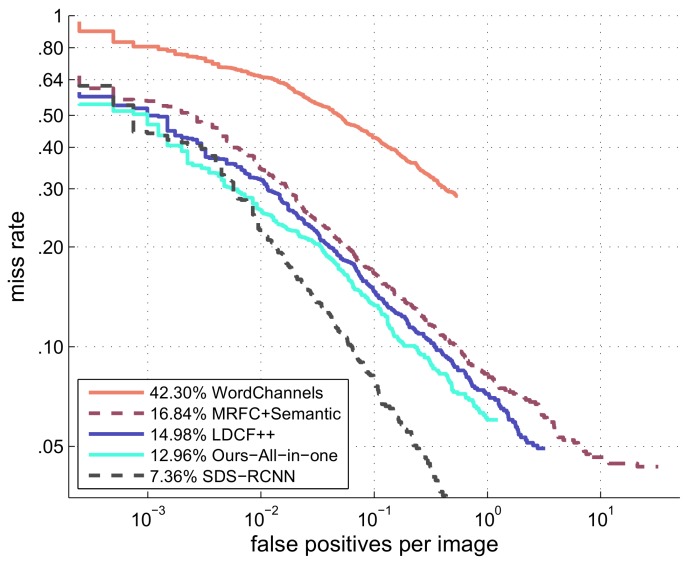
Comparison with the top methods and single scale feature maps-based methods on the Caltech dataset. Our method achieves the lowest miss rate for the whole FPPI range in all the non deep learning methods.

The miss rate vs. FPPI plot is shown in Figure 9. In this plot, for each FPPI point, the miss rate is the lower the better. From the figure we see that among non deep learning methods, our method achieves the lowest miss rate for the whole FPPI range. The MR values are given at the figure legend. According to the MR values, our method outperforms all the other non deep learning methods.

As in the KITTI experiment, we evaluate the impact of the five different components of our algorithm for Caltech dataset. The result is shown in Table 3. The trend is similar with that in the KITTI experiment. The fastest version (15.97%/27.72FPS) of our algorithm is by combining SAP, GPC and SGD, while the most accurate version is by combining SAP, SDT, motion features and GPC (12.62%/3.11FPS). Note that all the detector versions in the table achieve higher accuracy than MRFC + Semantic, including our baseline detector (only use SAP). Some of them are also faster than MRFC + Semantic.

**Figure 10:**
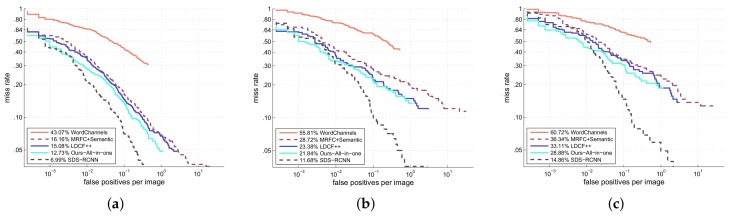
Evaluation results under conditions of small scale, atypical aspect ratio and partial occlusion. (**a**) Small scale (50 px≤h≤80 px); (**b**) Atypical aspect ratio (|w/h−0.41|≥0.1); (**c**) Patial occlusion (0–35% occluded).

We also evaluate our detector under conditions of small scale, atypical aspect ratio and partial occlusion, as shown in Figure 10. Small scale means the pedestrian height is between 50 px and 80 px. Aspect ratio is computed as w/h, where *w* and *h* is the pedestrian width and height respectively. According to [49], the distribution of w/h is centered at 0.41. Atypical aspect ratio is defined as |w/h−0.41|≥0.1. Partial occlusion means a pedestrian is occluded, but not more than 35%. From the figure we see that our detector also ranks the first among all the non deep learning methods under these conditions.

Though the accelerating strategies for sliding window classification can also be used in pyramid-based methods, the feature pyramid computing takes too much time and the accelerating effect is limited. For example, the second best non deep learning method LDCF++ [15] needs 0.65 s to construct a feature pyramid for a 480×640 image. Thus no matter what strategies are used in the sliding window classification, the detection speed could not be more than 1.54 FPS. While for our method (without motion channels), the feature map computing takes only 0.02 s using the same hardware, thus further speeding up is expected by using a better strategy in sliding window classification stage. For motion channels, though we do not construct pyramid either, even computing a single scale motion channels is very time consuming.

**Figure 11:**
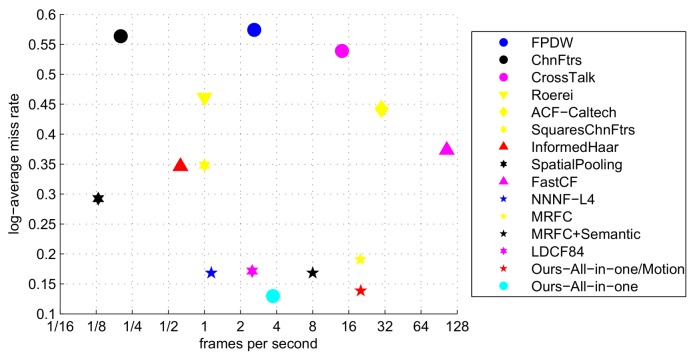
MR versus FPS on the Caltech Dataset.

In Figure 11, we show miss rate (the lower the better) versus FPS (the higher the better) of different algorithms which have given their detection time. We include two versions of our algorithm which with and without using motion channels. From the figure, we see both versions of our algorithm achieve high accuracy and competitive speed.

## 7. Conclusions

In this paper, we delve deep into the problem of multiscale pedestrian detection via single scale feature maps. Our work is inspired by MRFC, which achieves fast detection using single scale feature maps. We analysis two shortcomings of MRFC and propose scale-aware pooling and soft decision tree to tackle with them respectively. Because our solution leads to more computation cost in sliding window classification stage, we propose to use GPC and SGD to decrease the computation cost in this stage. Experiments on KITTI and Caltech dataset show our solution achieves high accuracy with fast detection speed.

## Figures and Tables

**Table 1:** AP on KITTI validation set using different β.

β	40	45	50	55	60
**AP**	68.02%	68.87%	69.25%	68.10%	67.81%

**Table 2:** AP and FPS on KITTI validation set under different setups of our method.

		Component for Speed	No Acceleration	+GPC	+SGD
	AP(%)/FPS				
Component for Accuracy					
**SAP**			67.17/1.54	67.85/3.85	67.29/6.85
**+SDT**			69.25/0.68	69.98/1.94	69.73/4.89
**+Motion**			69.98/0.61	70.53/1.65	70.07/3.40

**Table 3:** MR and FPS on Caltech test set under different setups of our method.

		Component for Speed	No Acceleration	+GPC	+SGD
	MR(%)/FPS				
Component for Accuracy					
**SAP**			16.45/9.62	15.89/18.36	15.97/27.72
**+SDT**			14.34/3.75	13.60/9.95	13.84/20.15
**+Motion**			13.08/2.29	12.62/3.11	12.96/3.72
